# Skin infection by *Mycobacterium farcinogenes*–*senegalense* group in an immunocompetent patient: a case report

**DOI:** 10.1186/s12879-022-07409-z

**Published:** 2022-05-10

**Authors:** An-Yu Cheng, Chih-Hung Lee

**Affiliations:** Department of Dermatology, Kaohsiung Chang Gung Memorial Hospital and Chang Gung University College of Medicine, Kaohsiung, Taiwan

**Keywords:** *Mycobacterium farcinogenes*–*senegalense* group, Skin infection, Cutaneous infection, Immunocompetent, Nontuberculous mycobacteria infection (NTM), Case report

## Abstract

**Background:**

*Mycobacterium farcinogenes*–*senegalense* group mostly cause bovine farcy, which rarely infect human beings. We reported one case of cutaneous *Mycobacterium farcinogenes*–*senegalense* group infection in an immunocompetent victim.

**Case presentation:**

A 66-year-old Taiwanese woman with hypertension developed tender nodules on her left dorsal foot for 2 months. Tissue culture identified *Mycobacterium farcinogenes*–*senegalense* group. The lesion was treated successfully with clarithromycin and sulfamethoxazole/trimethoprim, followed by surgical excision.

**Conclusions:**

*Mycobacterium farcinogenes*–*senegalense* group infection should be considered as a potential pathogen of skin infection in immunocompetent patients.

## Background

*Mycobacterium farcinogenes–senegalense* *group* is rapidly growing, non-tuberculous mycobacterium that causes bovine farcy. Mostly documented in sub-Saharan Africa, *Mycobacterium farcinogenes* and *Mycobacterium senegalense* result in chronic suppurative granulomas of skin and lymphatics in cattle. They are closely related to *Mycobacterium fortuitum and Mycobacterium houstonense.* In fact, *Mycobacterium farcinogenes–senegalense group* rarely affects human beings. [1]

## Case presentation

**Fig. 1:**
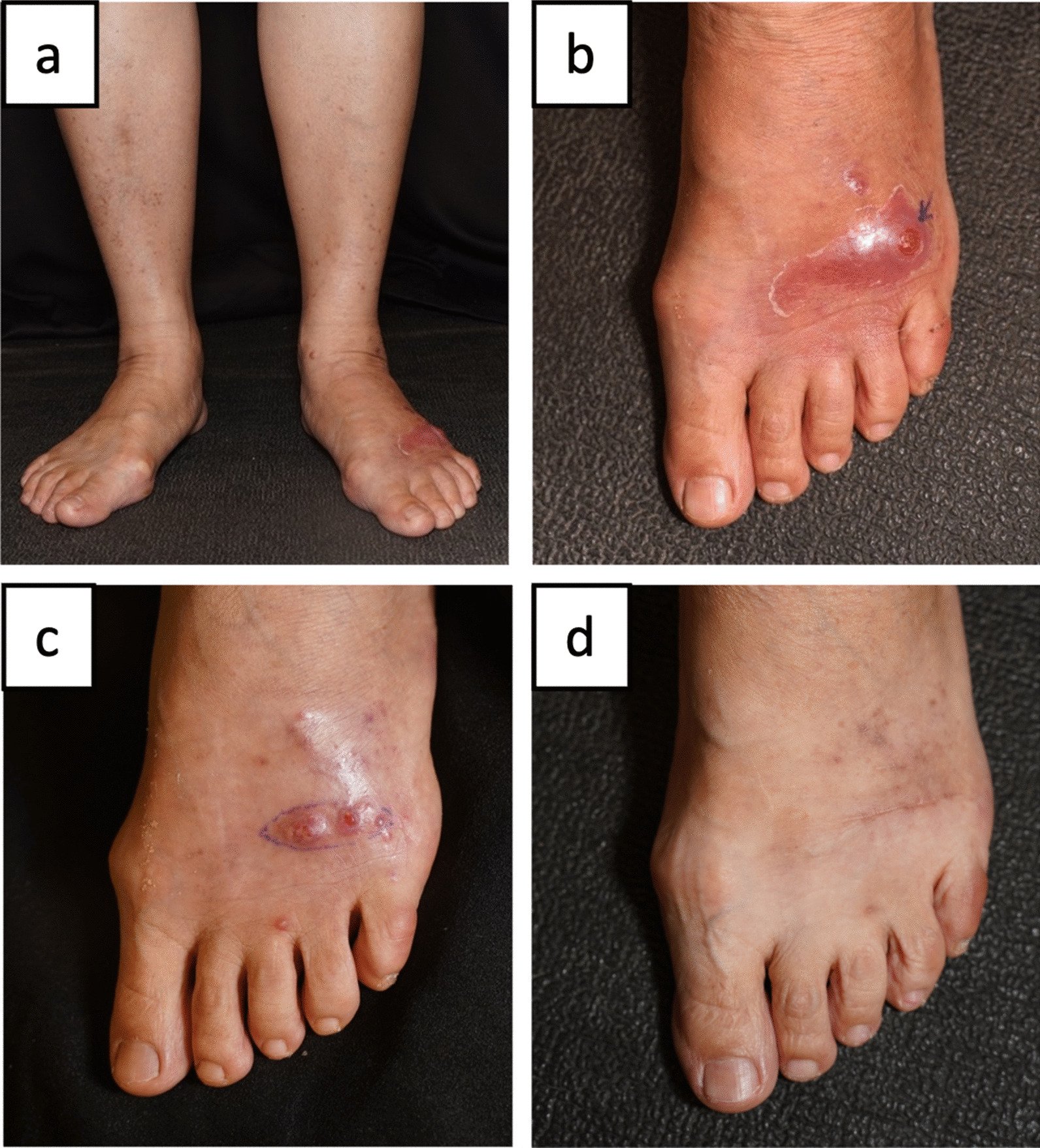
**a** One erythematous plaque with central ulceration on the left dorsal foot. **b** The close view of (**a**). **c** Lesion at week 10 was cured by surgery. **d** No recurrence was found for 8 months

A 66-year-old Taiwanese woman, who was free from immunocompromised conditions including diabetes mellitus or acquired immunodeficiency syndrome, developed enlarging tender nodules on her left dorsal foot for 2 months. Two months prior to this presentation, her left dorsal foot was traumatized by the spring of a trashed mattress. Physical exam showed reddened, indurated, and confluent nodules with pus discharge (Fig. 1). She was afebrile and she denied other constitutional symptoms. She initially tried acupuncture on the left lower leg for but failed. 10 days of empirical oral amoxicillin clavulanate (Augmentin^®^ 1250 mg/day) were administered after the sampling of the pus. Though the culture turned out to be negative, the lesion deteriorated. To exclude atypical infection, we performed skin biopsy for pathology exam, as well as fungal and mycobacterial culture.

**Fig. 2:**
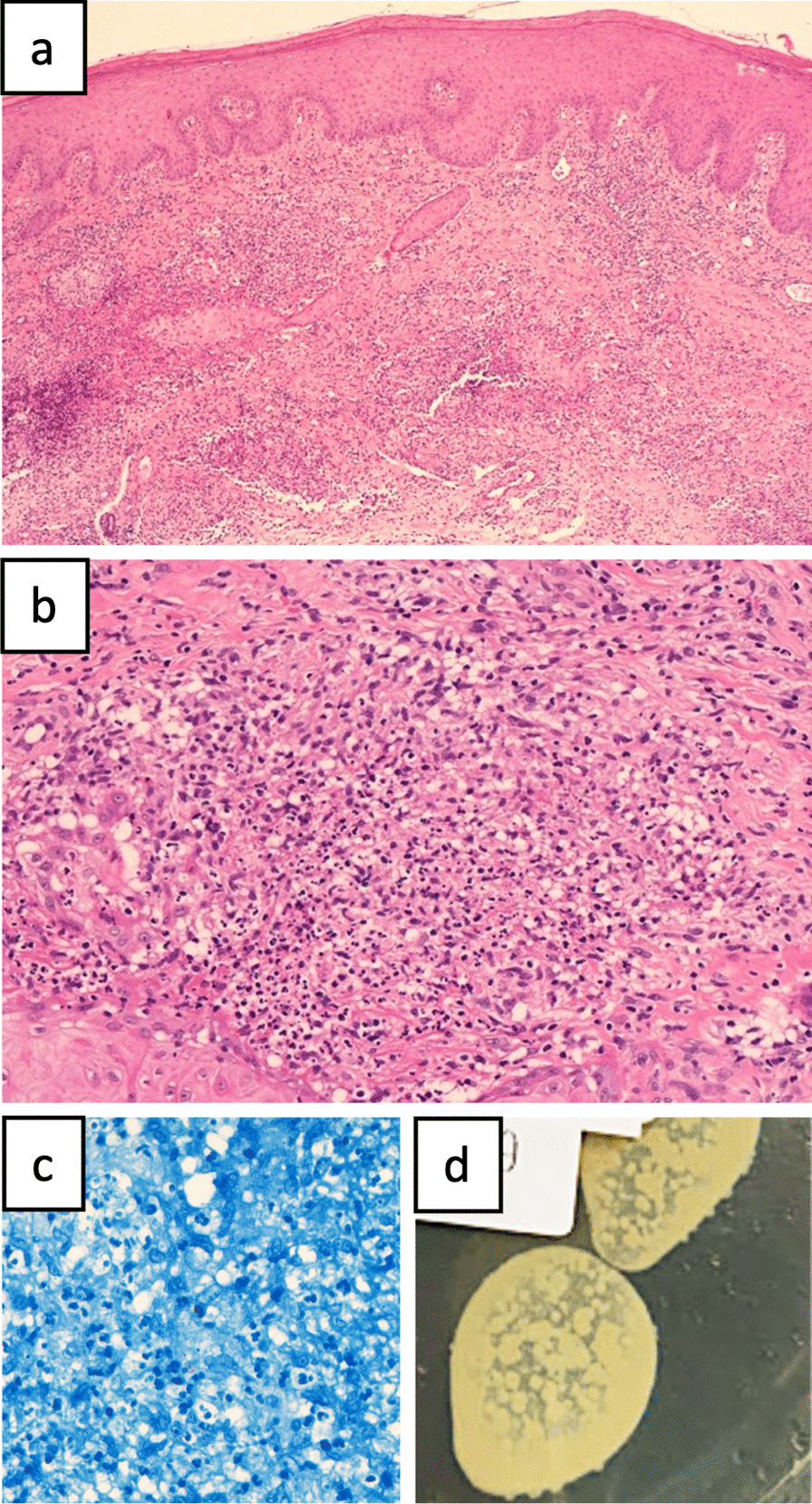
Histopathology exam revealed dermal granulomas composed of histiocytes and multinucleated giant cells with abscess. **a** Magnification: 50×; **b** magnification: 200×; **c** a few acid-fast bacilli identified by Ziehl–Neelsen stain, magnification: 400×. **d** The macroscopic view of the colony in the culture plate. (Middlebrook 7H11 Agar)

Microscopically, skin specimen demonstrated dermal suppurative granulomas composed of histiocytes and multinucleated giant cells. A few acid-fast bacilli were identified by Ziehl–Neelsen stain. 14 days after the culture, wheat-colored colonies developed (Fig. 2). Using Matrix assisted laser desorption ionization-time of flight mass spectrometry (MALDI-TOF MS; Bruker’s MALDI Biotyper, Bruker Libraries/Mycobacteria Library V2.0) to compare the extracted proteins from the colony to the reference, we successfully identified the pathogen to be the *Mycobacterium farcinogenes–senegalense group (MS score 1.94; MS score* 1.800–1.999 for species level for mycobacteria). After confirming the pathogen, the antibacterial regimens, featuring the combination of oral clarithromycin (1000 mg/day) and sulfamethoxazole/trimethoprim (Baktar^®^, 1960 mg/day), were administered for 2 months. Nevertheless, sulfamethoxazole/trimethoprim was held 2 weeks later due to hyperkalemia. The skin lesion resolved gradually thereafter and the residual lesion was eventually cured by surgical removal (Fig. 1d).

## Discussion and conclusions

MALDI-TOF MS detects the bacterial protein profile and identifies organisms based on database. It provides simple, fast, and reliable identification in most *Mycobacterium* species and is a potential alternative laboratory method in routine clinical care [2, 3].

We report a human skin infection by *Mycobacterium farcinogenes–senegalense*, which usually causes bovine farcy, in an immunocompetent woman after traumatic injury by rusty springs. The first human victim of *Mycobacterium senegalense* was a 49-year-old woman with non-Hodgkin’s lymphoma in Korea due to catheter-related bloodstream infection [4]. An immunocompetent 67-year-old man in Hong Kong had prosthetic infection of *Mycobacterium farcinogenes* after receiving total hip arthroplasty [5]. An immunocompetent 3-year-old North American girl got soft tissue infection of *Mycobacterium senegalense* after traumatic injury [6]. A North American 55-year-old immunocompetent male developed chronic osteomyelitis of* Mycobacterium senegalense* after traumatic ankle fracture [7]. Some following cases of osteomyelitis related to *Mycobacterium farcinogenes* were reported. [8, 9]. We summarized the above-mentioned cases in Table 1. We reported an immunocompetent case of *Mycobacterium farcinogenes–senegalense group* soft tissue infection in Asia, indicating that the infection is worldwide and not limited to immunocompromised patients. Due to its rarity, the consensus of the standard treatment is lacking. Treatment may include amikacin, cefoxitin, clarithromycin, ciprofloxacin, doxycycline, erythromycin, imipenem, trimethoprim/sulfamethoxazole, which is similar to the regimens for rapidly growing mycobacterium. Drug sensitivity test could be beneficial. Surgical excision could be considered in localized lesion in immunocompetent hosts.

**Table 1:** Cases of *Mycobacterium farcinogenes*–*senegalense* group

	Age/sex	Immune state	Country	Species	Infection site	trauma	Treatment course
Oh et al. [4]	49/F	Immunocompromised (non-Hodgkin’s lymphoma)	Korea	*M. senegalense*	Blood stream (catheter related)	None	Imipenem/cilastatin and amikacin then ciprofloxacin and doxycycline for 4 weeks
Wong et al. [5]	67/F	Immunocompetent	Hong Kong	*M. farcinogenes*	Prosthetic joint	Toal hip arthroplasty	Surgical removal of implant and debridement; ciprofloxacin and doxycycline intravenously for 6 weeks and then orally for 3 months
Talavlikar et al. [6]	3/F	Immunocompetent	America	*M. senegalense*	Soft tissue	Fish tank	Clarithromycin, trimethoprim/sulfamethoxazole and ciprofloxacin for 3 months
Maupin et al. [7]	55/M	Immunocompetent	America	*M. senegalense*	Bone	Traumatic ankle fracture (motor vehicle collision: from the driver’s seat into a pasture ditch)	Surgical removal of implant and debridement; imipenem, ciprofloxacin, and minocycline for 6 weeks, then imipenem, linezolid and azithromycin for 3 months, then linezolid, azithromycin, and doxycycline for another 3 months
Al Farsi et al. [8]	49/M	Immunocompromised (diabetes mellitus)	Oman	*M. farcinogenes*	Bone	Anterior cruciate ligament and medial meniscal repair	Surgical removal of implant and debridement; ciprofloxacin and doxycycline for 6 months
Kashihara et al. [9]	37/M	Immunocompetent	Japan	*M. farcinogenes*	Bone	Traumatic tibia and fibula fracture (concrete)	Surgical debridement; levofloxacin, amikacin, and rifampin for 12 months
Our case (2022)	66/F	Immunocompetent	Taiwan	*M. farcinogenes*–*senegalense* group	Skin	Trashed mattress	Clarithromycin and sulfamethoxazole/trimethoprim for 2 months followed by surgical excision (sulfamethoxazole/trimethoprim was discontinued at 2 weeks due to intolerance)

The conventional method in isolating bacterial strains from the tissue allows in vitro drug susceptibility test which is useful for clinical decision making. Using the MALDI-TOF method, we could only identify the strain as *Mycobacterium farcinogenes*–*senegalense* group. However, due to the institutional biosafety regulation, the sample was disposed once the lab data of MALDI-TOF was obtained. Therefore, we could not perform the drug susceptibility test at this point. Furthermore, the current database does not allow us to distinguish the species of these two closely related *Mycobacterium farcinogenes* and *Mycobacterium senegalense*.

In conclusion, we reported one rare *Mycobacterium farcinogenes–senegalense group* cutaneous infection in an immunocompetent patient in Taiwan. The lesion was partially resolved after 2 months of clarithromycin and finally cured by the surgical removal of the remaining. *Mycobacterium farcinogenes–senegalense group* infection should be considered as potential pathogen of skin infection.

## Data Availability

The datasets used and analyzed during the current study are available from the corresponding author on reasonable request.
